# Microbiome Analysis of Biofilms of Silver Nanoparticle-Dispersed Silane-Based Coated Carbon Steel Using a Next-Generation Sequencing Technique

**DOI:** 10.3390/antibiotics7040091

**Published:** 2018-10-22

**Authors:** Akiko Ogawa, Keito Takakura, Katsuhiko Sano, Hideyuki Kanematsu, Takehiko Yamano, Toshikazu Saishin, Satoshi Terada

**Affiliations:** 1 Department of Chemistry and Biochemistry, National Institute of Technology, Suzuka College, Suzuka 510-0294, Japan; h24c17@ed.cc.suzuka-ct.ac.jp; 2 D&D Corporation, Yokkaichi 512-1211, Japan; sano@ddcorp.co.jp; 3 Department of Material Science and Engineering, National Institute of Technology, Suzuka College, Suzuka 510-0294, Japan; kanemats@mse.suzuka-ct.ac.jp; 4 Department of Marine Technology, National Institute of Technology, Toba College, Toba 517-8501, Japan; yamano@toba-cmt.ac.jp (T.Y.); saishin@toba-cmt.ac.jp (T.S.); 5 Department of Applied Chemistry and Biochemistry, University of Fukui, Fukui 910-8507, Japan; terada@u-fukui.ac.jp

**Keywords:** biofilm, microbiomes, silver nanoparticles, silane-based coating, *Marinomonas*, *Anaerospora*

## Abstract

Previously, we demonstrated that silver nanoparticle-dispersed silane-based coating could inhibit biofilm formation in conditions where seawater was used as a bacterial source and circulated in a closed laboratory biofilm reactor. However, it is still unclear whether the microbiome of a biofilm of silver nanoparticle-dispersed silane-based coating samples (Ag) differs from that of a biofilm of non-dispersed silane-based coating samples (Non-Ag). This study aimed to perform a microbiome analysis of the biofilms grown on the aforementioned coatings using a next-generation sequencing (NGS) technique. For this, a biofilm formation test was conducted by allowing seawater to flow through a closed laboratory biofilm reactor; subsequently, DNAs extracted from the biofilms of Ag and Non-Ag were used to prepare 16S rRNA amplicon libraries to analyze the microbiomes by NGS. Results of the operational taxonomy unit indicated that the biofilms of Non-Ag and Ag comprised one and no phyla of archaea, respectively, whereas Proteobacteria was the dominant phylum for both biofilms. Additionally, in both biofilms, Non-Ag and Ag, *Marinomonas* was the primary bacterial group involved in early stage biofilm formation, whereas *Anaerospora* was primarily involved in late-stage biofilm formation. These results indicate that silver nanoparticles will be unrelated to the bacterial composition of biofilms on the surface of silane-based coatings, while they control biofilm formation there.

## 1. Introduction

A ship’s engine room has a plumbing system that includes a power unit, steam pipes, heat exchangers and fuel pipes. Generally, seawater is used in the ship’s cooling system; however, this can lead to biofouling. Biofouling is a series of bioprocesses where material surfaces are initially covered by conditioning films of non-organic polymers, followed by biofilm formation by microorganisms, such as bacteria and archaea, and then by macroorganisms, such as algae and balanoids, which adhere to the surface [1]. Biofouling reduces the efficiency of heat exchange in the cooling system, and it may at times result in the destruction of heat exchange pipes [2,3,4]. The key stage in biofouling is biofilm formation. Biofilms are composed of bacteria and their extracellular polymeric substrates and can cause microbially-influenced corrosion (MIC) of steel [5,6].

Previously, we demonstrated that a combination of silver nanoparticles and silane-based coating inhibited biofilm formation on the surface of pipes of water cooling systems that used seawater as a coolant [7]. However, the microbiome composition of biofilms is still unknown. In this study, we utilized a next-generation sequencing (NGS) technique to study microbiomes of biofilms on steels with silver nanoparticles-dispersed silane-based coating.

## 2. Materials and Methods

### 2.1. Specimens

A carbon steel JIS SS400 plate (Sakai Netsu-Giken, Tsu, Japan) was cut into coupons of an area of 10 × 20 mm^2^ (thickness, 1 mm) using a shearing machine (Komatsu Industries, Kanazawa, Japan). Each coupon was washed with acetone and then dried for 24 h in a desiccator before initiating the coating process. Silane-based coating solution was prepared as previously described [7]. Subsequently, silver nanoparticles (diameter, 100 nm; Sigma–Aldrich, St. Louis, MO, USA) were added to the silane-based coating solution. Next, the coating solution was filtered through a nylon mesh #110 (NBC Meshtec Inc., Hino, Japan) to remove any residues or impurities. The filtered Ag solution was then sprayed onto the surface of each SS400 coupon, which were subsequently incubated at 20 °C for 24 h.

### 2.2. Sampling Seawater

Seawater was collected in a sterile glass bottle (1 L, AGC Techno Glass, Haibara-gun, Japan) at a depth of 2 m on 7 November 2016. The sampling location was 1.6 km offshore in Ise Bay, Japan (34°31.20′ N, 136°48.36′ E). The seawater-filled bottle was covered using double-layered aluminum foil and stored in a refrigerator (4 °C–8 °C) until use.

### 2.3. Biofilm Formation Test

**Figure 1:**
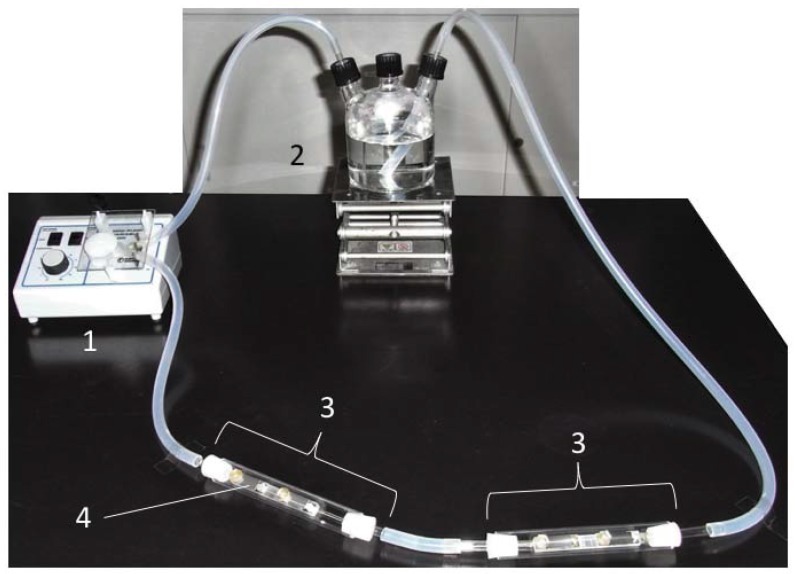
The closed laboratory biofilm reactor. 1: peristaltic pump; 2: culture bottle; 3: column; 4: sample holder. The seawater was circulated in a counterclockwise direction.

A laboratory biofilm reactor (LBR) comprising two polycarbonate columns (Sanplatec Corporation, Osaka, Japan), a three-necked, glass culture bottle and a peristaltic pump was used (Figure 1). All coupons were fixed in acryl holders (Sanplatec Corporation) using acryl screw pins (Sanplatec Corporation) with their coated surfaces facing upward. These holders were then placed in LBR columns, which were connected to silicone tubes (As One, Osaka, Japan). The silicone tubes were connected to the three-necked, glass culture bottle. After the assembly was sterilized using an autoclave (Tomy Seiko, Tokyo, Japan) at 121 °C for 15 min, the stored seawater (500 mL) was transferred to the sterile three-necked, glass culture bottle and was circulated by the peristaltic pump at a speed of dial 10 (Thermo Fisher Scientific, Waltham, MA, USA) at 20 °C for 139 h.

### 2.4. DNA Extraction from Seawater and Biofilms

A PowerSoil^®^ DNA isolation kit (MO BIO Laboratories, Carlsbad, CA, USA) was used for DNA extraction, both from the biofilms on the surface of each coupon and from microbes in the seawater. Stored seawater was filtered through a 0.1-μm polyethersulfone filter (Sartorius Japan, Tokyo, Japan). After filtration, the sediment on the filter was collected in PowerSoil^®^ Bead tubes (MO BIO Laboratories). Biofilms on the surface of each coupon were scratched using a sterile spatula, and the scratched biofilms were collected in PowerSoil^®^ Bead tubes. Then, 60 μL of Solution C1 was added to each tube, which was then inverted several times, secured to a bead crusher (TITEC, Koshigaya, Japan), vortexed at 4600 rpm for 1 min and placed on ice. Vortexing and cooling was repeated nine more times. Any DNA present in tubes was then purified according to a previously reported procedure [7]. The concentration of the purified DNA solution was measured using the Qubit fluorometer (Thermo Fisher Scientific) and a dsDNA high sensitivity (HS) assay kit (Thermo Fisher Scientific). 

### 2.5. 16S rRNA Gene-Based Bacterial Community Analysis

Bacterial and archaebacterial 16S rRNA genes were partially amplified using 16S rRNA V4 region primers: 515f (5′-ACACTCTTTCCCTACACGACGCTCTTCCGATCTGTGCCAG-CMGCCGCGGTAA-3′) and 806r (5′-GTGACTGGAGTTCAGACGTGTGCTCTTCCGATCT-GGACTACHVGGGTWTCTAAT-3′). The first sampled polymerase chain reaction (PCR) solution (total volume, 20 μL) comprised 2.0 μL of PCR buffer (TaKaRa BIO, Kusatsu, Japan), 1 U of ExTaq (TaKaRa BIO), 0.5 μM of primers (515f and 806r each), 0.2 mM of dNTP mixture, 2.0 μL of purified DNA sample and 12.2 μL of deionized distilled water (TaKaRa BIO). For PCR, the samples were initially subjected to 94 °C for 2 min, followed by 94 °C for 30 s, 50 °C for 30 s and 72 °C for 30 s (25 cycles) and finally by 72 °C for 5 min using a thermal cycler (TaKaRa BIO). At the tagging PCR step, each PCR amplicon was tagged for MiSeq Illumina sequencing using Index2 (a unique 8-bp sequence) and Index1 (5′-TCCTCTAC-3′), which was separately inserted into the 2ndF (5′-AATGATACGGCGACCACCGAGATCTACAC-Index2-ACACTCTTTCCCTACACGACGC-3′) and 2ndR primer (5′-CAAGCAGAAGACGGCATACGAGAT-Indexl-GTGACTGGAGTTCAGACGTGTG-3′). The tagging PCR solution (total volume, 20 μL) comprised 2 μL of the first PCR amplicon, 1 U of ExTaq, 2.0 μL of PCR buffer, 0.2 mM of dNTP mixture, 0.5 μM of 2ndF primer, 0.5 μM of 2ndR primer and deionized distilled water. The tagging PCR conditions were as follows: the initial step involved a treatment at 94 °C for 2 min; followed by a treatment at 94 °C for 30 s, 60 °C for 30 s and 72 °C for 30 s (8 cycles); and a final step of 72 °C for 5 min using a thermal cycler (TaKaRa BIO). After reaction completion, the concentration of all tagged PCR products was measured using a dsDNA HS assay kit and Qubit fluorometer. The quality of all tagged PCR products was assessed using a High Sensitivity NGS Fragment Analysis Kit (Advanced Analytical Technology, Ames, IA, USA) and Fragment Analyzer (Advanced Analytical Technology). Finally, all tagged PCR products were pooled in one tube, and NGS was performed using the MiSeq system (Illumina, San Diego, CA, USA). The 16S rRNA gene library was pre-processed and analyzed using a combination of USEARCH [8,9], bioinformatics software package QIIME [10] and FASTX-Toolkit [11], a fast processing tool. Complete tag-matching sequences were extracted from forward- and reverse-sequence raw data files using the fastq_barcode_splitter.pl script, and then, the primer sequences were removed. Extracted sequences were trimmed, and specific sequences, which had a quality score of <20 and length of <40 bp, were discarded using sickle tools [12]. Trimmed forward and reverse sequences with a merged length of 260 bases, reading length of 230 bases and minimum overlap size of 10 bases were merged using the FLASH software [13,14]. Next, merged sequences were filtered from 246 bases to 260 bases, then chimeric sequences were checked using the identify_chimeric_seqs.py script and uchime algorithm of USEARCH against reference sequences of 97% operational taxonomic units (OTU) using the Greengenes database [15]. After removing chimeric sequences from the merged sequences, non-chimeric sequences were clustered based on 97% identity, and an OTU table was created using the pick_de_novo_otus.py script. The Greengenes database was used for taxonomically assigning the bacteria. Raw data files have been deposited in the NCBI Sequence Read Archive and are awaiting an accession number.

## 3. Results and Discussions

We compared the microbiomes of stored seawater samples (Seawater), of biofilms on the surface of non-dispersed silane-based coating samples (Non-Ag) and of biofilms on the surface of silver nanoparticle-dispersed silane-based coating samples (Ag). At the phylum level, three archaea were detected in Seawater; two archaea were detected on the biofilms of Non-Ag; and no archaea were detected on the biofilms of Ag (Table 1). Crenarchaeota contains hyperthermophilic and acidophilic bacteria [16], whereas Euryarchaeota contains methanogens and halophiles [17]. The content of archaea in Seawater was approximately 2%, whereas that of archaea on the biofilms of Non-Ag was approximately 0.1%. The outstanding difference in the microbiomes of biofilms of Non-Ag compared with that of biofilms of Ag was that no archaea were observed on the biofilms on Ag. These results indicate that marine archaea do not prefer silane-based coating surfaces for attachment and growth; moreover, silver nanoparticles will enhance the effect of silane-based coating killing archaea.

**Figure 2:**
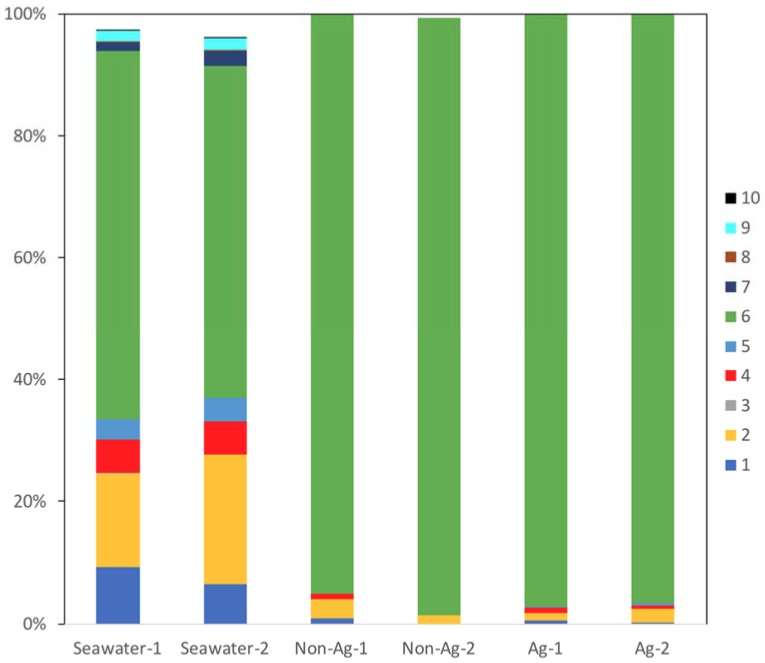
Main bacterial phyla detected in the seawater (Seawater), biofilms of silane-based coating samples (Non-Ag) and biofilms of silver nanoparticles-dispersed silane-based coating samples (Ag). Unassigned and archaeal OTUs were removed. When all samples showed phyla with an abundance of <0.1%, the phyla were excluded from the bacterial percentages. 1: Actinobacteria; 2: Bacteroidetes; 3: Chloroflexi; 4: Cyanobacteria; 5: Planctomycetes; 6: Proteobacteria; 7: SAR406; 8: SBR1093; 9: Verrucomicrobia; 10: ZB3.

In all samples, the most common bacterial phylum identified was *Proteobacteria* (#6 in Figure 2), and over 90% of all organisms identified on the biofilms of Non-Ag and Ag belonged to this phylum. This result showed one possibility that MIC may occur in either Non-Ag or Ag because Proteobacteria comprises many MIC-related bacteria such as *Pseudomonas*, sulfate-reducing bacteria, *Acidithiobacillus* and *Gallionella*. Meanwhile, the proportion of Actinobacteria (#1 in Figure 2), Bacteroidetes (#2 in Figure 2), Cyanobacteria (#4 in Figure 2) and Planctomycetes (#5 in Figure 2) had markedly reduced on the biofilms of Non-Ag and Ag compared with Seawater. Huttunen-Saarivirta et al. reported significantly higher biofilm formation on Grade EN 1.4162 stainless steel than that on five other common stainless steels [18]. Moreover, Actinobacteria and Proteobacteria were the main phyla on the biofilms of EN 1.4162 and those of five other common stainless steels. However, the biofouling tendency differed from the corrosion behavior among these six stainless steels [18]. Yu et al. reported that Proteobacteria, Actinobacteria and Bacteroidetes were detected on the biofilms formed on several types of water-distribution pipes and that a plastic-based pipe was suitable for water distribution due to its low biofilm-forming potential and microbial diversity [19]. Considering these reports, both Ag and Non-Ag will have low potential to form biofilms.

**Figure 3:**
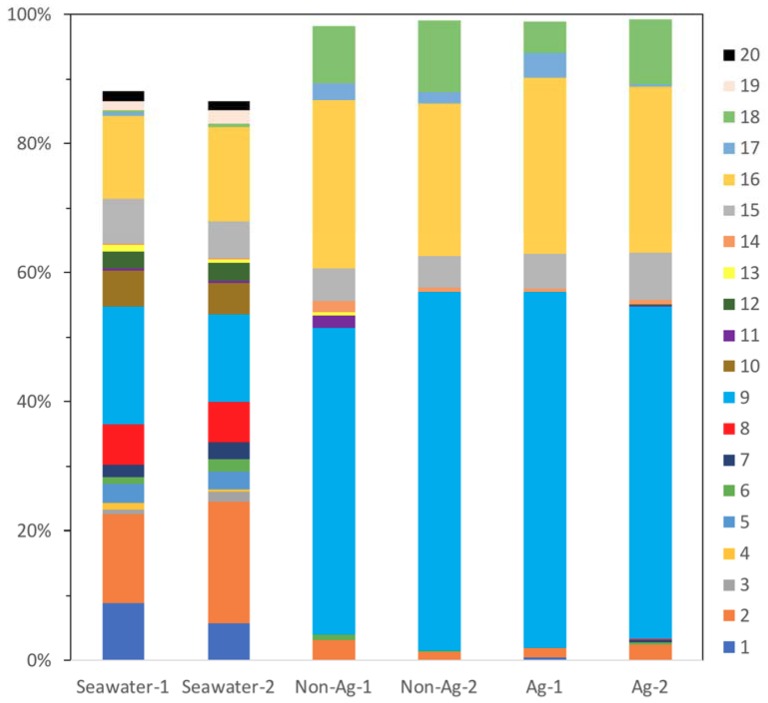
Main bacterial orders detected in the seawater (Seawater), biofilms of silane-based coating samples (Non-Ag) and biofilms of silver nanoparticles-dispersed silane-based coating samples (Ag). Unassigned and archaeal OTUs were removed. When all samples showed orders with an abundance of <1.0%, the orders were excluded from the bacterial percentages. 1: Acidimicrobiales; 2: Flavobacteriales; 3: Rhodothermales; 4: Cryptophyta; 5: Stramenopiles; 6: Synechococcales; 7: unknown order of Pla3; 8: unknown order of Alphaproteobacteria; 9: Rhodobacterales; 10: Rickettsiales; 11: Burkholderiales; 12: Rhodocyclales; 13: Sva0853; 14: Campylobacterales; 15: Alteromonadales; 16: Oceanospirillales; 17: Pseudomonadales; 18: Vibrionales; 19: Arctic96B-7; 20: unknown order of Pedosphaerae.

At the order level, for Seawater, there were three major bacterial orders: Flavobacteriales of Bacteroidetes (OTU, 14% and 19%, #2 in Figure 3), Rhodobacterales of Alphaproteobacteria (OTU, 14% and 18%, #9 in Figure 3) and Oceanospirillales of Gammaproteobacteria (OTU, 13% and 15%, #16 in Figure 3), respectively. Conversely, biofilms of Non-Ag and Ag had two major bacterial orders: Rhodobacterales, which comprised approximately 50% of the microbiomes, and Oceanospirillales, which comprised approximately 25% of the microbiomes. Bacteroidetes are well known as a main marine bacterial group present across many oceans. They play a vital role in degrading organic compounds in the ocean [20]. In this study, all Seawater, Non-Ag and Ag contained Flavobacteriales of Bacteroidetes; they mainly occupied 14–19% in Seawater; however, Non-Ag and Ag had a low proportion of this order of 1–3%, which means Flavobacteriales had difficulty proliferating on the surface of Non-Ag and Ag. Therefore, the biofilm formation conditions provided by Non-Ag and Ag would not be suitable for Flavobacteriales growth. The order Rhodobacterales contains the family Rhodobacteraceae, whose members are key biofilm formers at the initial stage of biofilm formation in seawater [21]. Besides, Alteromonadales (#15 in Figure 3) and Oceanospirillales have been reported in young biofilms and with potential tolerance toward polysaccharide biodegradation and carbohydrate metabolism in biofilms [22]. In this study, the biofilms of Ag and Non-Ag were rich in Rhodobacterales and Oceanospirillales, indicating an early, not matured, stage of these biofilms. However, Rhodospirillales, whose members are known as marine biocorrosion bacteria [22], was only detected in Seawater (OTU, 0.6% and 0.9%). Additionally, higher proportions of Vibrionales (#18 in Figure 3), which include *Vibrio*, were detected on the biofilms of Non-Ag (OTU, 8.8% and 11.2%) and Ag (OTU, 5.0% and 10.2%) compared with those in Seawater (OTU, 0.5%). The members of *Vibrio* are considered to be corrosion-protective bacteria [22]. These results indicate the possibility that biofilms of Non-Ag and Ag did not contain MIC-related bacteria. As discussed in the previous section, Proteobacteria comprises many MIC-related bacteria, but OTUs of the order level show that the members of Proteobacteria identified on the biofilms of Non-Ag and Ag rarely cause MIC; however, these bacteria are highly related to biofilm formation. The biofilm formation test involved a six-day culture, which was twice the time period used in the previous experiment where Ag was shown to inhibit biofilm formation [7]. Unfortunately, we could not procure sufficient DNA samples to analyze the next generation in the previous three-day culture. Thus, we extended the culture period for the current experiment and obtained sufficient DNA from both the biofilms of Ag and Non-Ag. However, extending the culture period may affect the microbial composition on the biofilm of Ag, i.e., increased number of initial biofilms of Ag may comprise different bacteria. 

It is still unclear as to whether the biofilms of Ag and Non-Ag comprise similar biofilm-forming bacteria. In order to estimate the presence of biofilm-forming bacteria in this experiment, three abundant, order-level bacterial groups were summarized (Table 2, Table 3 and Table 4). On the biofilms of both Non-Ag and Ag, *Anaerospora* of Rhodobacteraceae was the dominant genus, followed by *Marinomonas* of Oceanospirillaceae and an unknown genus of the Vibrionaceae of Vibrionales (not *Vibrio*)*. Marinomonas* is an aerobic bacterial genus; Vibrionaceae is a facultative aerobic bacterial family; and *Anaerospora* is an anaerobic bacterial genus, although only *Anaerospora hongkongensis* is registered as a member of *Anaerospora* [23,24]. When considering the oxygen requirement of bacteria, anaerobic bacteria would be more abundant than aerobic bacteria on the biofilms of Non-Ag and Ag. Extracted DNA from the biofilms was derived from living bacterial cells and extracellular DNA [25], which can be released from either living or dead cells that may no longer be present on the biofilms. Hence, we assumed that OTUs would help determine the history of biofilm-related bacteria. Our current biofilm formation test was performed in a closed LBR where dissolved oxygen concentration was estimated to be high during the early culture period, but very poor during the late culture period because stored seawater was used and no additional oxygen was supplied. Based on the presence of Oceanospirillales in the young biofilms, *Marinomonas* were considered to be the dominant biofilm-forming bacteria in early-stage biofilm formation in the LBR. Later, *Anaerospora* dominated as the main biofilm-forming bacteria as the dissolved oxygen concentration decreased during the culture period. In the bacterial phylum section, we considered that MIC might occur in either Non-Ag or Ag because abundant bacteria of biofilms of Non-Ag and Ag were Proteobacteria comprising many MIC-related bacteria such as *Pseudomonas*, sulfate-reducing bacteria, *Acidithiobacillus* and *Gallionella*. Indeed, the abundant members of Proteobacteria of biofilms of Non-Ag and Ag were *Anaerospora* and *Marinomonas*, which were biofilm-forming bacteria, but not reported for MIC. On the other hand, sulfate-reducing bacteria such as *Desulfovibrio*, *Acidithiobacillus* and *Gallionella* had undetectable levels there; however, *Pseudomonas* was slightly detected in the biofilm of Ag (OTU, 0.01% and 0.99%). These results show that both Non-Ag and Ag had biofilms formed on the surface, but they rarely progressed to MIC.

We have already reported that the amount of biofilm of Ag was significantly less than that of Non-Ag [7]; however, what kinds of bacteria formed biofilm was unclear. Therefore, we approached revealing the microbiota of biofilms of Ag and Non-Ag. Comparing the OTU percentages of the biofilm of Ag with that of the biofilm of Non-Ag, Figure 2 and Figure 3 demonstrate that the microbiota composition of biofilm of Ag was almost the same as that of the biofilm of Non-Ag. Therefore, we presumed that the microbiota composition of biofilms formed on silane-based coating did not change whether silver nanoparticles were dispersed in the coating or not; whereas, silver particles in the silane-based coating would control the attachment, growth and quorum sensing of bacteria on the surface, resulting in a small amount of biofilm.

## 4. Conclusions

In our previous study, we found that a silver nanoparticle-dispersed silane-based coating effectively inhibited biofilm formation. Here, we attempted the analysis of microbiomes on biofilms formed on the coatings using an NGS technique. No archaea phyla were detected on the biofilms of Ag, whereas only one archaea phylum was detected on the biofilms of Non-Ag. Proteobacteria were the dominant bacterial phylum on the biofilms of both Non-Ag and Ag. Comparing OTUs of the biofilms on Ag with those of the biofilms on Non-Ag, no distinct difference was noted in bacterial orders, but biofilm-forming bacterial orders and a biocorrosion-protective bacterial order were found to be present in both biofilms. In addition, members of *Marinomonas* were the main biofilm-forming bacteria under aerobic conditions, but were replaced by those of *Anaerospora* under anaerobic conditions. Moreover, the addition of silver nanoparticles did not affect the microbiome of biofilms on silane-based coating, while it decreased the amount of biofilm on it.

## Figures and Tables

**Table 1:** Percentage abundance of operational taxonomic units of archaea.

Seawater-1	Seawater-2	Non-Ag-1	Non-Ag-2	Ag-1	Ag-2	Phyla
0.58	0.54	0.12	0.00	0	0	Crenarchaeota
1.20	1.90	0	0	0	0	Euryarchaeota
0.02	0.03	0	0.11	0	0	Parvarchaeota

**Table 2:** Percentage abundance of operational taxonomic units of Rhodobacterales. -: unknown.

Seawater-1	Seawater-2	Non-Ag-1	Non-Ag-2	Ag-1	Ag-2	Families	Genera
5.29	3.79	4.77	4.51	3.86	3.30	Rhodobacteraceae	Other
5.50	4.11	3.32	2.53	3.67	1.66		-
0.29	0	0	0	0	0		*Amaricoccus*
6.15	4.33	34.86	44.79	47.15	40.30		*Anaerospora*
0.06	0.04	0	0.01	0.01	0		*Loktanella*
0	0.02	0	0	0	0		*Octadecabacter*
0.01	0.01	0.02	0.42	0.01	0.03		*Paracoccus*
0.89	1.18	4.42	3.31	0.46	6.05		*Pseudoruegeria*
0.03	0.02	0.01	0	0	0		*Rhodobacter*
0	0	0.01	0.01	0.01	0.01		*Rhodovulum*
0.01	0	0	0.01	0.01	0.01		*Roseivivax*
0.86	0.59	0	0	0	0	Rhodospirillaceae	-
0.04	0	0	0	0	0		*Magnetospirillum*

**Table 3:** Percentage abundance of operational taxonomic units of Oceanospirillales. -: unknown.

Seawater-1	Seawater-2	Non-Ag-1	Non-Ag-2	Ag-1	Ag-2	Families	Genera
0	0	1.80	0.65	0.81	1.09	Oceanospirillaceae	Other
1.72	1.70	1.89	2.58	3.72	3.92		-
0	0	0	0.26	0	0.15		*Amphritea*
0.28	0.32	21.76	19.56	18.97	19.60		*Marinomonas*
0	0	0	0	0	0.27		*Neptunomonas*
0	0.01	0	0	0	0		*Oceanospirillum*
0	0	0.64	0.30	2.40	0.06		*Oleispira*
0	0	0	0.31	0.56	0.41		-
0.01	0.02	0	0	0	0	SUP05	-

**Table 4:** Percentage abundance of operational taxonomic units of Vibrionales. -: unknown.

Seawater-1	Seawater-2	Non-Ag-1	Non-Ag-2	Ag-1	Ag-2	Families	Genera
0	0	0	0.01	0	0.01	Pseudoalteromonadaceae	-
0.03	0.04	1.21	1.68	0.24	0.90		*Pseudoalteromonas*
0.41	0.43	7.30	9.47	3.27	9.17	Vibrionaceae	Other
0	0	0.01	0.02	0	0.01		-
0.07	0.01	0.05	0	1.45	0		*Photobacterium*
0.01	0.02	0.21	0.03	0.01	0.08		*Vibrio*
